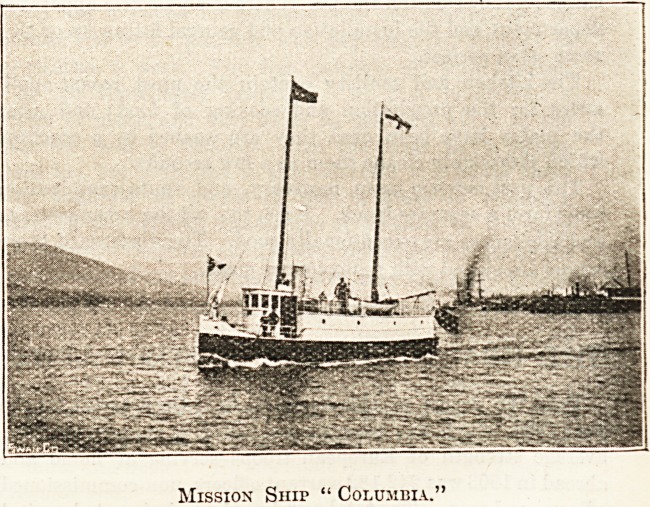# Mission and Hospital Ship in Canadian Waters

**Published:** 1905-08-12

**Authors:** 


### MISSION AND HOSPITAL SHIP IN CANADIAN WATERS.

BY A CORRESPONDENT.

On May 29th a small vessel was consecrated in Victoria Harbour, British Columbia, which is unique in its double character (as far as the Pacific coast is concerned), being both a missionary and hospital ship.

The scheme is the outcome of the energy of a Canadian clergyman, the Rev. J. Antle, who realised the needs of some 3,000 men engaged in logging and mining on the East coast of Vancouver Island, on the mainland of British Columbia, and on the intervening islands. For these there has hitherto been no definite provision made for spiritual and bodily needs when ill. As a result of much strenuous effort Mr. Antle has collected sufficient funds to pay for the mission vessel, *The Columbia*, and for present working expenses. Having had a nautical training before studying for holy orders, he himself designed the handy little craft, which he can also navigate. The ship is of four tons only; her cost was about $4,000, and she is worked by gasoline with auxiliary sails. There are

Mission Ship "Columbia."

two boats attached, one of which contains a gasoline engine, which will enable the doctor to go ashore or follow up the vessel if necessary. In the central cabin there is sitting accommodation for 50 to 60 persons to attend divine service. This cabin also contains a small organ, a library, and two hospital beds, cupboards, and lockers, as well as a complete medical and surgical outfit. The chaplain's and doctor's cabins, the kitchen, and forecastle complete the interior of a very compact little craft.

Dr. Hutton, who was recently in charge of the Garfield Memorial Hospital, Washington, D.C., has most generously offered his services for a time gratuitously. As he understands engineering and the chaplain acts as skipper, one able-bodied seaman and the cook complete the ship's crew.

Connected with the above mission, there will be a small hospital with six beds, now in course of construction at the Hastings Sawmill, Vancouver, to be transmitted in sections to Rock Bay. This is a small settlement, occupying a central position in the district to be handled by *The*

*Columbia* Mission, having a logging camp of 200 men, a large store and an hotel. The Victorian Nursing Order of Canada will supply a graduate nurse, a probationer and an orderly. Accident cases can therefore be skilfully handled from first to last.

It is hoped that this mission will eventually become self-supporting, but definite help is yet needed to establish it, and donations, however small, will be gladly received by Rev. J. Antle, Mission Ship *Columbia*, Vancouver, B.C., Canada.

## Figures and Tables

**Figure f1:**